# Maternal Smoking during Pregnancy and Fetal Organ Growth: A Magnetic Resonance Imaging Study

**DOI:** 10.1371/journal.pone.0067223

**Published:** 2013-07-03

**Authors:** Devasuda Anblagan, Nia W. Jones, Carolyn Costigan, Alexander J. J. Parker, Kirsty Allcock, Rosanne Aleong, Lucy H. Coyne, Ruta Deshpande, Nick Raine-Fenning, George Bugg, Neil Roberts, Zdenka Pausova, Tomáš Paus, Penny A. Gowland

**Affiliations:** 1 Sir Peter Mansfield Magnetic Resonance Centre, University of Nottingham, Nottingham, Nottinghamshire, United Kingdom; 2 Nottingham University Hospitals NHS Trust, Nottingham, Nottinghamshire, United Kingdom; 3 Clinical Research Imaging Centre, Queens Medical Research Institute, Edinburgh, United Kingdom; 4 Rotman Research Institute, University of Toronto, Toronto, Ontario, Canada; 5 Division of Obstetrics & Gynaecology, School of Clinical Sciences, University of Nottingham, Nottingham, Nottinghamshire, United Kingdom; 6 Research Institute of the Hospital for Sick Children, University of Toronto, Toronto, Ontario, Canada; 7 Universite de Montreal, Montreal, Quebec, Canada; 8 School of Psychology, University of Nottingham, Nottingham, Nottinghamshire, United Kingdom; University of Southampton, United Kingdom

## Abstract

**Objective:**

To study whether maternal cigarette smoking during pregnancy is associated with alterations in the growth of fetal lungs, kidneys, liver, brain, and placenta.

**Design:**

A case-control study, with operators performing the image analysis blinded.

**Setting:**

Study performed on a research-dedicated magnetic resonance imaging (MRI) scanner (1.5 T) with participants recruited from a large teaching hospital in the United Kingdom.

**Participants:**

A total of 26 pregnant women (13 current smokers, 13 non smokers) were recruited; 18 women (10 current smokers, 8 nonsmokers) returned for the second scan later in their pregnancy.

**Methods:**

Each fetus was scanned with MRI at 22–27 weeks and 33–38 weeks gestational age (GA).

**Main outcome measures:**

Images obtained with MRI were used to measure volumes of the fetal brain, kidneys, lungs, liver and overall fetal size, as well as placental volumes.

**Results:**

Exposed fetuses showed lower brain volumes, kidney volumes, and total fetal volumes, with this effect being greater at visit 2 than at visit 1 for brain and kidney volumes, and greater at visit 1 than at visit 2 for total fetal volume. Exposed fetuses also demonstrated lower lung volume and placental volume, and this effect was similar at both visits. No difference was found between the exposed and nonexposed fetuses with regards to liver volume.

**Conclusion:**

Magnetic resonance imaging has been used to show that maternal smoking is associated with reduced growth of fetal brain, lung and kidney; this effect persists even when the volumes are corrected for maternal education, gestational age, and fetal sex. As expected, the fetuses exposed to maternal smoking are smaller in size. Similarly, placental volumes are smaller in smoking versus nonsmoking pregnant women.

## Introduction

It is well recognised that adverse environmental conditions can affect fetal development. Although some of these effects may be adaptive in the short term, many can have long-term consequences for the health of the individual in subsequent childhood and even adulthood [1]. Cigarette smoke contains a multitude of different compounds that may be harmful to the developing fetus; although 20% of pregnant smokers quit smoking before the initial prenatal visit, approximately 15–20% of pregnant women smoke tobacco during pregnancy despite a strong public-health campaign over the last few decades [2].

Prenatal exposure to maternal cigarette smoking (PEMCS) is associated with a wide range of obstetric complications including increased risk of perinatal mortality, miscarriage, gestational bleeding, placental abruption, placenta praevia, as well as neonatal and early-childhood disorders, such as sudden infant death syndrome (SIDS) and respiratory diseases [3]–[5]. In particular, PEMCS is known to be the most common cause of intrauterine growth restriction (IUGR) [5]. Despite being born smaller at birth, individuals exposed prenatally to maternal smoking are at higher risk for obesity and obesity-associated disorders, such as cardiovascular and diabetes outcomes, beginning in childhood or adolescence [6], [7]. There is also evidence suggesting that PEMCS is associated with a number of adverse outcomes related to the offspring’s behaviour [8] and brain development [9]–[11].

In this study, we recruited pregnant women who either smoked (cases) or did not smoke (controls) cigarettes and investigated fetal growth using magnetic resonance imaging (MRI) considering the whole fetus, the placenta, and a number of major organs (brain, kidneys, lungs and liver). Tobacco smoking is frequently associated with epiphenomena, such as risky behavior, co-abuse of other substances, poor prenatal care, and low socioeconomic status, which will also exert adverse effects on the developing fetus. Therefore, we attempted to match the cases and controls for socioeconomic status, age, body mass index (BMI), and parity.

## Materials and Methods

This research was conducted at the University of Nottingham with the approval of the Nottingham Research Ethics Committee, in accordance with principles of Good Clinical Practice. All volunteers gave informed written consent to participate in the study.

### Research Participants

Data were collected from 26 healthy pregnant women recruited from the Queen’s Medical Centre, Nottingham: 13 current smokers and 13 non-smokers. Only 10 current smokers and 8 non-smokers returned for the second visit and so only their data were analyzed. Women were eligible to participate if they had a viable, singleton pregnancy and were aged ≥18 years. At recruitment, smokers and non-smokers were matched for age (±3 years: range: 18–36), parity, BMI, and education level (grouped by a. less than 5 General Certificates of Secondary Education (GCSEs: school leaving qualification taken by 16 year olds in the UK), b. more than 5 GCSEs, c. A-levels (standard method of assessing admission to undergraduate studies in the UK and is generally taken at 18 years old), d. university degree). All of these data were collected through questionnaires administered by their obstetricians.

At each clinic visit, participants were asked to record their smoking status, and if they were smokers, the quantity, frequency and tobacco brand used. Gestational age was established using the crown-rump length based on ultrasound, carried out at the time of the routine dating scan. Two MRI scans were performed at gestational ages of 24 (±2) weeks and 35 (±2) weeks.

### MRI Acquisition

All scans were conducted using a 1.5 tesla Philips Achieva MRI system using either a 5-element SENSE cardiac or 4-element SENSE torso receive coil, depending on the woman's size. Women lay on their right side in the decubitus position to avoid compression of the vena cava, and scans were performed with a specific absorption rate of <2.0 W kg^−1^.

Three sequences were acquired to give a range of contrasts and resolutions for different organs: (1) HASTEbody: Half Fourier Single Shot Turbo Spin-Echo (HASTE); 123 slices in 147 seconds, TE = 120 ms, 0.78×0.78×6.00 mm^3^ voxels, (2) HASTEbrain: to provide higher spatial resolution particularly over the brain (34 slices in 36 seconds, TE = 120 ms, 0.59×0.59×4.50 mm^3^ voxels) and (3) bFFE: Balanced Fast Field Echo (130 slices in 167 seconds, TR = 5.8 ms, TE = 2.3 ms, flip angle = 70°, 0.78×0.78×6.00 mm^3^ voxels). All sequences were acquired in three orthogonal blocks of images, without breath holding. This over-acquisition of the data allowed the effects of motion and artifacts to be excluded or averaged out.

### Image Analysis

Volumes (cm^3^) were estimated by drawing a region of interest (ROI) around the organ of interest in a series of images of contiguous slices and then summing the areas of the ROI in each slice and multiplying by the slice thickness. Two approaches were used to measure areas, with a different, blinded operator undertaking each. The first was manual segmentation of every slice using semi-automatic edge detection (Analyze 9.0, Mayo Clinic, Rochester, MN, USA), which was used for smooth, high contrast objects (fetal kidneys and lungs, total fetus and placenta on bFFE, and lungs on HASTEbrain). The second was the Cavalieri stereological method, which was applied in combination with point counting (EasyMeasure software, University of Liverpool, UK) [12]–[15]. A 20×20 voxel grid was superimposed on the image, and the fraction of the grid points lying within the ROI gave the fraction of the whole area of the image representing the ROI. This segmentation technique was used to measure fetal brain and liver on the HASTEbrain data. If, for any participants, the HASTEbrain images were seriously affected by motion or signal non-uniformity artifacts, then HASTEbody scans were used instead. Volumes were measured on all relevant slices from at least two of the orthogonal blocks of images acquired from each sequence and averaged. These two estimates differed by approximately 0–10% from the mean. Finally, the volume of each organ as a percentage of the total fetal volume was calculated (% volume for each organ).

Fetal length (cm) was calculated from the sum of the length of the fetal spine (measured in two curved sections), thigh, lower leg and skull height (crown to base of the brain stem) on bFFE and HASTE images using Analyze 9.0. In order to measure the length of the spine, the multi-slice images were resampled obliquely. Fetal Volume Index (FVI) was calculated as the ratio of Fetal Volume to Fetal Length.

### Statistical Methods

Independent sample t-tests were used to examine group differences in maternal age, maternal BMI and maternal parity. A chi-square test was used to examine group differences in maternal education.

A univariate analysis of co-variance (ANCOVA) was completed for birth weight and gestational age at birth (weeks). Each independent analysis included one between-subject variable of exposure to maternal cigarette smoking. Three covariates were included in these analyses: fetal sex, maternal education (education was re-coded as an ordinal variable: 0 = 0 GCSEs; 1 = <5 GCSEs; 2 = >5 GCSEs/A-levels/degree) and maternal age. Post-hoc comparison analyses were completed with a Bonferroni correction for multiple comparisons.

A mixed-model ANCOVA was completed for the following fetal variables of interest: brain volume, kidney volume, lung volume, liver volume, total fetal volume, placental volume, FVI, percentage brain volume, percentage kidney volume, percentage lung volume and percentage liver volume. Each independent analysis included one within-subject variable of visit (24 weeks, 35 weeks) and one between-subject variable of exposure to maternal cigarette smoking. Four covariates were included in all analyses: fetal sex, maternal education (education was re-coded as an ordinal variable: 0 = 0 GCSEs; 1 = <5 GCSEs; 2 = >5 GCSEs/A-levels/degree), gestational age at Visit 1 and the difference in gestational age between Visits 1 and 2. Post-hoc comparison analyses were completed with a Bonferroni correction for multiple comparisons.

## Results

**Figure 1:**
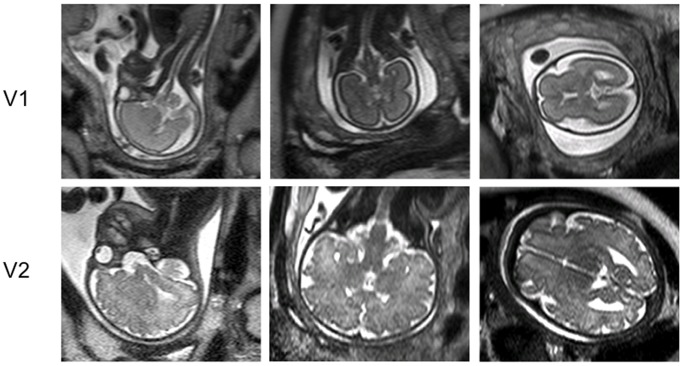
MR Images through the fetal brain in three orthogonal planes (HASTEbrain sequences), 24 weeks (V1) and 35 weeks (V2).

Typical images of the fetal brain are shown in Figure 1.

Table 1 shows the demographics and the delivery details for all mothers. Smoking mothers smoked 9±4 cigarettes per day. Although participants were matched at recruitment, due to drop out, smoking mothers who completed the study were significantly younger than non-smoking mothers [by 4.8 years, p = 0.041], although they did not differ in parity, height, or adiposity. Similarly, smoking mothers were less educated than non-smoking mothers, but this difference was not significant (p = 0.056). At delivery, babies of smoking mothers (exposed) did not differ from babies of non-smoking mothers (non-exposed) with respect to their birth weight (p = 0.124). The exposed babies also did not differ in their gestational age at delivery (p = 0.868). There were no cases of pre-eclampsia in either group. However, there were two cases of pregnancy-induced hypertension (one in each group). Pregnancy induced hypertension is defined as blood pressure >140/90 mmHg observed on two separate occasions (>4 hours apart) and in the absence of proteinuria. There were two cases of fetal growth restriction in the exposed group; fetal growth restriction is defined as birth weight below the 10th centile corrected for gestational age at delivery, infant’s sex, and whether a first-born or not [16].

**Table 1:** Demographics and the delivery details for all mothers.

Characteristic	Normal (n = 8)			Smoker (n = 10)		
	Mean	SD	Range	Mean	SD	Range
**Maternal age (years)**	29.5	4.9	20–33	24.7	4.2	18–29
**Weight (kg)**	71.1	11.2	59–92	65.9	13.8	55–88
**Height (cm)**	167.5	6.5	162–176	166.4	4.1	160–175
**BMI**	24.5	3.7	20–30	23.4	5.5	18–33
**Parity**	0.5	0.8	0–2	1.2	1.2	0–3
**Cigarette smoked per day**	N/A	N/A	N/A	9	4	4–15
**Gestational age at delivery (weeks+days)** *	39.9	1.4	37.3–41.9	39.8	1.4	37.7–41.6
**Birth weight (g)** *	3547	597	2880–4220	3036	582	2170–4140
**Education**	1 with 0 GCSEs, 1 with <5 GCSEs, 4 with >5 GCSEs, 1 with A-Levels and 1 with a degree			2 with 0 GCSEs, 5 with <5 GCSEs and 3 with >5 GCSEs		
**Delivery**	2 Emergency Caesarean, 1 elective Caesarean deliveries			2 Ventouse deliveries		

* Note that the estimated means and standard deviations for gestation at delivery and birth weight were corrected for fetal sex, maternal education (education was re-coded as an ordinal variable: 0 = 0 GCSEs; 1 = <5 GCSEs; 2 = >5 GCSEs/A-levels/degree), and maternal age as derived from the ANCOVA (IBM SPSS Statistics Version 20, NY, USA).

**Figure 2:**
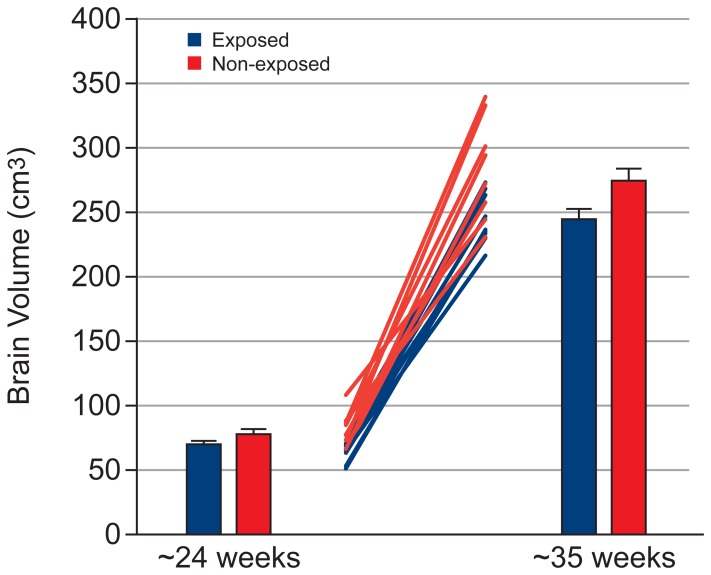
Brain growth data showing group results at both visits and individual data.

**Figure 3:**
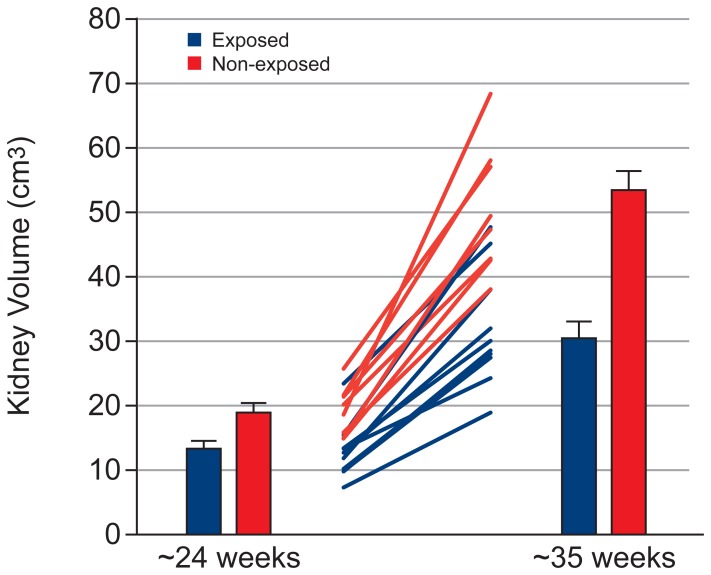
Kidney growth data showing group results at both visits and individual data.

**Figure 4:**
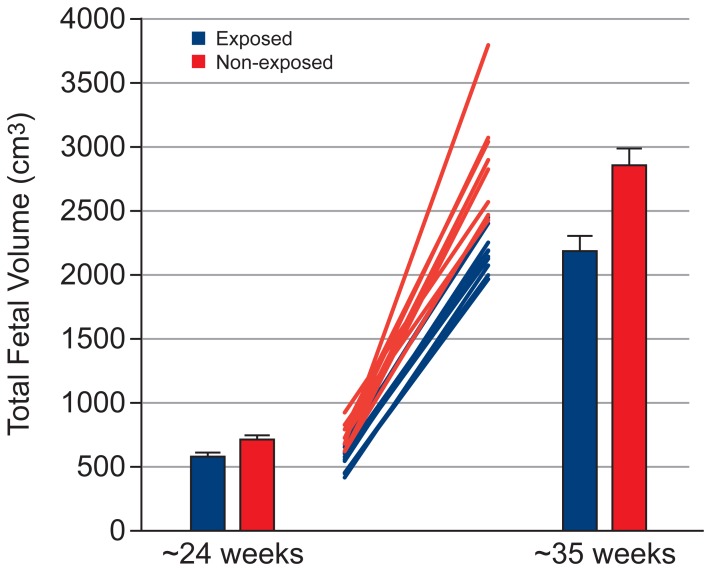
Fetal growth data showing group results at both visits and individual data.

**Figure 5:**
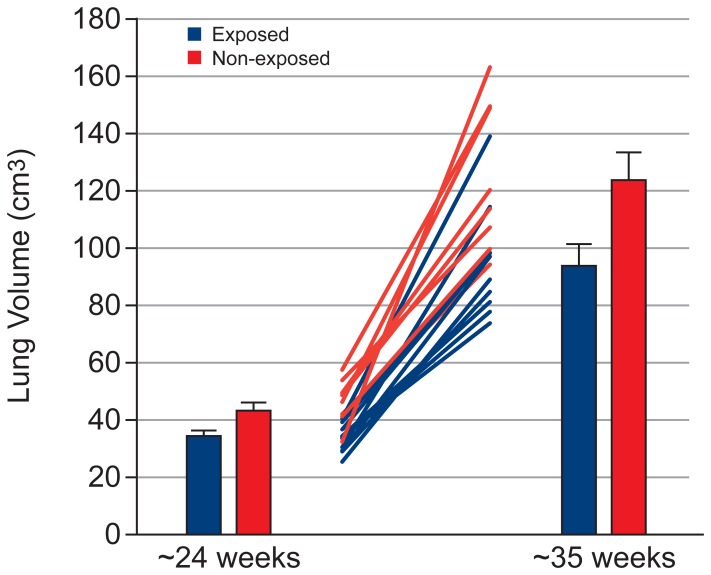
Lung growth data showing group results at both visits and individual data.

**Figure 6:**
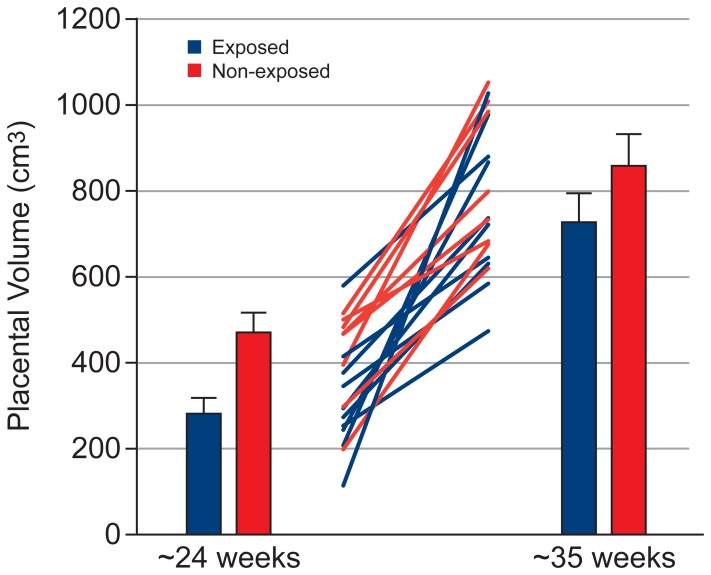
Placental growth data showing group results at both visits and individual data.

Table 2 shows the effect of exposure on the volume of fetal organs, assessed using a mixed model ANCOVA. Tables 3 and 4 show the mean and standard error for fetal organ volumes, placenta volume and total fetal volume at Visit 1 and 2, respectively. Exposed fetuses showed lower brain volume (Figure 2, p = 0.02), with this effect being greater at Visit 2 (Cohen’s d = 1.31) than Visit 1 (Cohen’s d = 0.85; but note that the exposure × visit interaction failed to reach significance [p  =  0.079]). Exposed fetuses showed lower kidney volume (Figure 3, exposure × visit interaction: p  =  0.001) and total fetal volume (Figure 4, exposure × visit interaction: p  =  0.009) compared with non-exposed fetuses at both Visit 1 (kidney: p = 0.028, Cohen’s d = 1.31; total fetal volume: p = 0.002, Cohen’s d = 2.07) and Visit 2 (kidney: p<0.001, Cohen’s d = 2.70; total fetal volume: p = 0.005, Cohen’s d = 1.80). Exposed fetuses also demonstrated lower lung volume (Figure 5, p = 0.018) and placental volume (Figure 6, p = 0.022), and this effect was similar at both visits. No difference was found between the exposed and non-exposed fetuses with regards to liver volume (p > 0.1). Exposed fetuses also showed lower percentage kidney volume (p = 0.04) and percentage brain volume (p = 0.051). No differences were found between the exposed and non-exposed fetuses with regard to percentage lungs and liver volume. Finally, exposed fetuses showed a lower FVI (p = 0.047) compared with non-exposed fetuses.

**Table 2:** The effect of exposure on the volume of fetal organs was assessed using a mixed model ANCOVA.

Volumes	p-values	
	Exposure (Main Effect)	Exposure×Visit
Brain	0.020	0.079
Lung	0.018	0.166
Kidney	<0.001	0.001
Liver	0.106	0.152
Placenta	0.022	0.685
Total Fetal	0.003	0.009
Percentage Brain	0.051	0.473
Percentage Lung	0.564	0.735
Percentage Kidney	0.04	0.595
Percentage Liver	0.867	0.919

Each independent analysis included one within-subject variable of visit and one between-subject variable of exposure to maternal cigarette smoking, with four covariates.

**Table 3:** The mean and standard error for fetal organ volumes, placenta volume and total fetal volume at Visit 1.

	Visit 1				
Volumes	Non Exposed		Exposed		p-values
	Mean (cm^3^)	SEM (cm^3^)	Mean (cm^3^)	SEM (cm^3^)	
Brain	76.737	3.236	69.018	2.824	0.129
Lung	44.719	2.362	35.470	2.062	0.020
Kidney	18.925	1.554	13.255	1.357	0.028
Liver	62.285	5.104	53.075	3.650	0.205
Placenta	469.719	46.126	280.283	40.257	0.016
TotalFetal	719.206	21.657	593.963	18.901	0.002

The mean and standard error were corrected for fetal sex, maternal education (education was re-coded as an ordinal variable: 0 = 0 GCSEs; 1 = <5 GCSEs; 2 = >5 GCSEs/A-levels/degree), gestational age at Visit 1 and the difference in gestational age between Visits 1 and 2.

**Table 4:** The mean and standard error for fetal organ volumes, placenta volume and total fetal volume at Visit 2.

	Visit 2				
Volumes	Non Exposed		Exposed		p-values
	Mean (cm^3^)	SEM (cm^3^)	Mean (cm^3^)	SEM (cm^3^)	
Brain	277.506	8.504	246.453	7.422	0.028
Lung	125.300	9.617	94.694	8.393	0.051
Kidney	53.500	3.051	30.451	2.662	<0.001
Liver	234.143	19.464	189.354	13.920	0.116
Placenta	862.388	74.402	728.033	64.934	0.241
Total Fetal	2871.135	133.065	2202.110	116.132	0.005

The mean and standard error were corrected for fetal sex, maternal education (education was re-coded as an ordinal variable: 0 = 0 GCSEs; 1 = <5 GCSEs; 2 = >5 GCSEs/A-levels/degree), gestational age at Visit 1 and the difference in gestational age between Visits 1 and 2.

## Discussion

This study has confirmed that PEMCS is associated with a reduced volume of multiple fetal organs, and that in some cases this effect can be detected as early as mid-pregnancy (∼22 weeks).

Whilst women continue smoking in pregnancy, this work, along with other studies focusing on the effect of PEMCS, addresses the recent request from the Department of Health directed towards the National Institute for Health and Clinical Excellence (NICE) to provide public health guidance on interventions aimed at stopping smoking in pregnancy [17]. Many of these interventions focus on providing information about the risks of PEMCS to the unborn child.

Nicotine and carbon monoxide have been identified as two major compounds in cigarette smoke that may have harmful effects on the developing fetus during pregnancy. They are known to cross the placenta and can be detected in fetal circulation, amniotic fluid, and the breast milk of smoking mothers. There is evidence that the concentration of nicotine in fetal circulation is 15% higher than that found in maternal circulation [18] and that the concentration of nicotine in the amniotic fluid is 88% higher than in the maternal plasma [2], [18]. Nicotine can affect the fetus in many ways: (a) vasoconstriction of the uteroplacental vasculature leading to uteroplacental underperfusion and a reduction in nutrient and oxygen flow to the fetus [19]; (b) suppression of the mother’s appetite leading to poor nutrition and energy intake and resulting in reduced energy supplied to the fetus; and (c) alterations in the cellular growth and activity of the central and peripheral nervous system [20]. Carbon monoxide binds with hemoglobin to form carboxyhemoglobin resulting in depletion in oxygen supplied to fetal tissue and hypoxia-ischemia. Studies in animal models demonstrate evidence of histopathological changes in the fetal lungs, liver, kidneys and placenta in response to cigarette smoke [21], [22].

The reduced brain volume in PEMCS fetuses is consistent with our previous findings. We have previously demonstrated a smaller volume of the corpus callosum in female adolescents with PEMCS [10] and a smaller (by ∼14%) total area of the cerebral cortex in female adolescents with a particular variant of the *KCTD8* gene [9]. It has been suggested that variations in fetal brain growth associated with prenatal exposure to maternal cigarette smoking (human studies) or nicotine (experimental studies) might be due to increased apoptosis of both progenitor cells and post-mitotic neurons [9], [20].

The effect of PEMCS on reducing fetal lung growth was previously observed in rat fetuses, and is potentially the result of a reduction in airway size [23]. Recent studies have detailed microscopic changes in the lungs of fetal rats with prenatal exposure to nicotine [24], [25] or cigarette smoke [22], [24] and there is evidence of increased risk of reduced respiratory function in infants exposed to maternal smoking during pregnancy [26]. Future studies could use MR techniques to study lung maturation as well as lung growth in developing fetuses [27].The data also show that PEMCS particularly affects the growth of fetal kidneys and kidney growth rate; this result persists after correcting for total fetal volume, indicating that the reduction in fetal kidney volume is not a result of fetal growth restriction. This result agrees with previous reports of decreased kidney weight in offspring of pregnant rats exposed to nicotine [28]. Similarly, it has been reported that there is a positive association between kidney malformation and maternal smoking [29]. Whilst the interaction of carbon monoxide and nicotine in kidney organogenesis is not clear, maternal smoking can cause disturbances in the interaction between the ureteric bud and the metanephric mesenchyme. Moreover, numerous immature renal corpuscles have been observed in fetuses exposed to PEMCS, which is suggestive of disturbed kidney development [22].

The placental volume data demonstrated that smaller placental volumes are associated with PEMCS. Several studies have documented a negative effect of PEMCS on placental structure, particularly in the placentae of heavy smokers [21], [30]. This indicates that further in vivo studies of placental function in PEMCS are required. There is also evidence of reduced volume density of the fetal vessels in the terminal villi and reduced exchange area in the placentae of women who smoke [31], [32], possibly leading to decreased uterine blood flow in the placenta due to a vasoconstriction mechanism [33]. Further studies are in progress and will involve relating placental diffusion in PEMCS to placental weight and morphology.

### Limitations of Our Study

MRI was used to study the effect of PEMCS on fetal organ growth and fetal volume. Due to the challenges in recruiting this particular, matched population, the study was small, reducing the power for detecting subtle differences. Furthermore, this is an observational study and, as such, it does not allow us to draw any causal inferences about the reported associations between PEMCS and organ growth. Fetal imaging is challenging for three reasons: unpredictable fetal motion, variations in the position of the fetus which means that the scan orientation must be adjusted for each subject, and a large field of view (especially third trimester) which can cause artifacts including wrap-around artifacts and signal inhomogeneities. These potential limitations were generally overcome by routinely oversampling the data and acquiring it in orthogonal orientations. Measurements of each organ were made on volume data sets acquired in at least two orientations (sagittal, coronal or transverse), with the individual averaged measurements for each organ varying by approximately 0–10%. These measurements were made by a trained operator but the manual segmentation approach used will inevitably have introduced some additional systematic and random errors into the results. Two different methods of drawing ROIs were used here. Computer-assisted manual segmentation has shown comparable coefficients of error of <5% for brain volumetry [34] and up to 10% for various fetal organs [35]. The second was the Cavalieri method for which a coefficient of error has previously been reported to be less than 5% for the fetal brain [13], and 10% for the fetal lung and liver [12]. Fully automated ROI drawing would reduce some of these errors further.

### Conclusion

In conclusion, the results of the present study demonstrate that intrauterine exposure to cigarette smoking is associated with impaired fetal and fetal organ growth, particularly in the kidney and brain.
